# A Training-Free Paradigm for Data-Scarce Maritime Scene Classification Using Vision-Language Models

**DOI:** 10.3390/s26082549

**Published:** 2026-04-21

**Authors:** Jiabao Wu, Yujie Chen, Wentao Chen, Yicheng Lai, Junjun Li, Xuhang Chen, Wangyu Wu

**Affiliations:** 1 Merchant Marine College, Shanghai Maritime University, Shanghai 201306, China; wujiabao@stu.shmtu.edu.cn (J.W.); 202310122056@stu.shmtu.edu.cn (Y.C.); 202310121287@stu.shmtu.edu.cn (Y.L.); lijj@shmtu.edu.cn (J.L.); 2 School of Computer Science and Engineering, Huizhou University, Huizhou 516007, China; xuhangc@hzu.edu.cn; 3 School of Computer Science, University of Liverpool, Liverpool L69 3DR, UK; wangyu.wu@liverpool.ac.uk

**Keywords:** vision-language models, optical spaceborne sensors, maritime domain awareness, intelligent sensing networks, training-free inference, sensor data processing

## Abstract

Maritime Domain Awareness (MDA) relies heavily on data acquired from high-resolution optical spaceborne sensors; however, processing this massive quantity of sensor data via traditional supervised deep learning is severely bottlenecked by its dependency on exhaustively annotated datasets. Under extreme data scarcity, conventional architectures suffer severe performance degradation, rendering them impractical for time-critical, zero-day deployments. To overcome this barrier, we propose a training-free inference paradigm that leverages the extensive pre-trained knowledge of Large Vision-Language Models (VLMs). Specifically, we introduce a Domain Knowledge-Enhanced In-Context Learning (DK-ICL) framework coupled with a Macro-Topological Chain-of-Thought (MT-CoT) strategy. This approach bridges the perspective gap between natural images and top–down optical sensor imagery by translating expert remote sensing heuristics into a strict, step-by-step reasoning pipeline. Extensive evaluations demonstrate the substantial efficacy of this framework. Armed with merely 4 visual exemplars per category as in-context triggers, our MT-CoT augmented VLMs outperform traditional models trained under identical scarcity by over 38% in F1-score. Crucially, real-world case studies confirm that this zero-gradient approach maintains robust generalization on unannotated, out-of-distribution coastal clutters, achieving performance parity with data-heavy networks trained on 50 times the data volume. By substituting massive human annotation and GPU optimization with scalable logical deduction, this paradigm establishes a resource-efficient foundation for next-generation intelligent maritime sensing networks.

## 1. Introduction

MDA serves as the backbone for global trade monitoring, maritime security, and marine environmental protection [1]. With the continuous advancements in Earth observation technologies, high-resolution optical spaceborne sensors have become the indispensable modality for monitoring maritime activities. Over the past decade, the field of remote sensing data processing has been dominated by supervised deep learning paradigms [2]. Architectures ranging from Convolutional Neural Networks (CNNs) to Vision Transformers (ViTs) have achieved phenomenal success in vessel detection and fine-grained classification based on sensor data [3]. However, this architectural triumph is heavily predicated on a fundamental assumption: the availability of massive, exhaustively annotated datasets.

In modern MDA, Earth observation sensors, ranging from multispectral optical cameras to synthetic aperture radars (SAR) mounted on satellites, generate unprecedented volumes of surveillance data. However, interpreting this complex sensor data efficiently remains a critical challenge. This inherent data dependency exposes the Achilles’ heel of conventional supervised learning in practical, engineering-oriented MDA applications: extreme data scarcity [4]. In time-critical, real-world maritime scenarios (such as emergency disaster response, search and rescue operations, or zero-day reconnaissance in uncharted waters), the prospect of acquiring, annotating, and training on thousands of optical sensor images is fundamentally impractical. This creates an unacceptable latency in deployment. When constrained to an extreme few-shot regime, traditional CNNs inevitably suffer from catastrophic overfitting and severe performance degradation. Furthermore, they frequently exhibit alarming false-positive rates when confronted with out-of-distribution coastal clutters, severely limiting their practical applicability. To overcome this bottleneck, the integration of advanced sensor networks with VLMs offers a transformative approach. By utilizing VLMs as intelligent processing hubs for raw sensor data, we can empower the shipping and maritime domain with rapid, autonomous, and highly resilient monitoring capabilities.

Recently, the advent of VLMs has catalyzed a paradigm shift in the broader computer vision community. Pre-trained on Internet-scale image–text pairs, VLMs encapsulate extensive prior world knowledge and sophisticated cross-modal reasoning capabilities [5,6]. This breakthrough introduces a compelling hypothesis for remote sensing engineering: Can we bypass the prohibitive costs and delays of gradient-based fine-tuning, and leverage the zero-shot or few-shot inference capabilities of off-the-shelf VLMs to enable rapid deployment in data-scarce MDA scenarios?

Migrating general-domain VLMs directly to optical satellite sensor data, however, encounters a profound epistemic gap. VLMs are predominantly pre-trained on feature spaces captured by ground-based natural optical sensors (e.g., consumer cameras) [7]. In stark contrast, spaceborne remote sensing sensors dictate a top–down, overhead perspective where maritime targets are heavily compressed in pixel space, lacking the fine-grained geometric side profiles familiar to the models [8]. Due to this fundamental perspective disparity, directly applying off-the-shelf VLMs and generic prompting techniques often yields suboptimal results, as the models lack the specialized operational context required to correctly interpret overhead scenes.

To unlock the full implementation potential of VLMs for remote sensing, it is essential to bridge this perspective gap through enhanced contextual engineering. While step-by-step reasoning mechanisms, such as Chain-of-Thought (CoT), are powerful catalysts for complex visual comprehension, they must be explicitly tailored to the physical characteristics of spaceborne sensors [9]. By integrating domain-specific interpretation rules and providing representative few-shot visual exemplars as cognitive anchors, a specialized reasoning chain can significantly enhance model sensitivity and boost target recall, ensuring high reliability for practical deployment.

Driven by the critical need for robust, engineering-oriented solutions in real maritime scenarios, this paper advocates for a paradigm shift toward macro-semantic maritime scene understanding. Utilizing the classical MAritime SATellite Imagery (MASATI) dataset and real-world out-of-distribution imagery, we propose a training-free Domain Knowledge-Enhanced In-Context Learning (DK-ICL) framework. Most importantly, we introduce a Macro-Topological Chain-of-Thought (MT-CoT) strategy. By explicitly injecting expert remote sensing knowledge into the reasoning pipeline, we successfully align the VLM’s natural prior with the overhead perspective, transforming abstract optical sensor data into highly structured, actionable semantic rationales.

The primary contributions of this paper, emphasizing both methodological innovation and practical implementation potential, are summarized as follows:

- A Training-Free Paradigm for Rapid Zero-Day Deployment: We pioneer the evaluation of off-the-shelf VLMs for macro-scene classification. By utilizing strictly frozen parameters, our DK-ICL framework bypasses the massive sunk costs of data annotation and GPU training. It achieves highly competitive scene understanding using a minimal fraction of traditional training data, demonstrating immense implementation potential for instantaneous deployment in urgent maritime missions.
- Operationalizing Expert Knowledge via MT-CoT: To bridge the perspective gap between natural and spaceborne sensor imagery, we propose a novel Macro-Topological CoT (MT-CoT). By injecting expert interpretation rules and few-shot visual exemplars, we explicitly teach the VLM how to execute step-by-step spatial reasoning. This operationalizes abstract domain knowledge into a practical engineering pipeline, which substantially increases target recall and stabilizes the classification process.
- Robustness and Practical Applicability in Real-World Scenarios: Our comprehensive analyses reveal that the VLM, augmented by our MT-CoT strategy, exhibits superior robustness in distinguishing complex coastal infrastructure (hard negatives) from actual vessels. It substantially outperforms traditional few-shot models, proving its high reliability and practical applicability for operational deployment in cluttered, real-world maritime environments.

The rest of this paper is organized as follows. Section 2 reviews related work. Section 3 introduces the research framework. Section 4 reports the data and main empirical results. Section 5 discusses the key findings. Section 6 concludes and suggests future research directions.

## 2. Literature Review

### 2.1. Traditional Deep Learning for Maritime Scene Classification

Over the past decade, supervised deep learning has significantly advanced MDA. Traditional maritime classification and vessel detection have heavily relied on CNNs, which extract hierarchical spatial features from complex environments. For instance, optimized CNN architectures have been widely utilized to establish robust baselines for vessel classification [10], and specific two-stage algorithms like Faster R-CNN have been adapted for accurate ship recognition in maritime transportation [11]. Furthermore, Vision Transformers (ViTs) have recently been introduced to the remote sensing domain to capture global dependencies. Researchers have successfully applied ViTs to classify sea surface phenomena [12], improved scene classification performance by combining ViTs with contrastive learning [13], and achieved fine-grained ship classification by fusing CNNs with Swin Transformers [14].

Despite these architectural triumphs, the success of these conventional deep learning paradigms is heavily predicated on massive, exhaustively annotated datasets. Data scarcity remains a fundamental bottleneck in deep learning, severely limiting model generalization [15]. In the remote sensing domain, data annotation is notoriously expensive and time-consuming [16], often forcing researchers to rely on error-tolerant strategies with extremely low annotation rates [17]. This shortage of tools and labeled data is particularly prominent in complex marine conditions [18,19]. Consequently, the community has increasingly focused on few-shot and low-shot learning to mitigate training instability. Numerous lightweight and adaptive networks have been proposed for maritime object detection under few-shot scenarios [20,21], open-water monitoring [22], and even underwater adaptive detection [23]. To handle incremental categories and cross-domain challenges, specific few-shot methodologies like Meta-RCNN and adaptive lightweight models have been developed [24,25]. Enhanced Vision Transformers have also been specifically tailored to address limited labeled samples in maritime target recognition [26].

Furthermore, when constrained by limited data or operating in near-shore areas, traditional supervised models frequently exhibit alarming false-positive rates. Complex coastal backgrounds and clutter frequently cause high false alarms for ship target detection [26]. Deep learning models easily mistake coastline infrastructure or background elements for ships, leading to severe detection confusion [27]. Additionally, fuzzy visual segments at the junction of ships and the shore consistently lead to false positives in bounding box predictions [28]. Therefore, the remote sensing community urgently requires a paradigm shift towards highly data-efficient methodologies capable of mitigating false positives in complex maritime backgrounds without relying on massive annotations.

### 2.2. Vision-Language Models and the Reasoning Gap in Remote Sensing

Recently, the advent of VLMs has catalyzed a revolution in the broader artificial intelligence landscape. Pre-trained on Internet-scale multimodal corpora, foundational models possess profound prior world knowledge and sophisticated cross-modal semantic reasoning capabilities. A defining characteristic of these models is their ability to perform complex visual tasks through zero-shot or few-shot inference. This breakthrough paves the way for open-vocabulary visual-understanding tasks, aligning effectively with real-world applications without the need for exhaustive fine-tuning [29,30]. Consequently, researchers have rapidly begun exploring the application of these models for zero-shot scene classification [31] and multi-sensor image comprehension in the remote sensing domain [32]. Furthermore, Multimodal Large Language Models (MLLMs) are actively being leveraged to construct high-quality, global-scale vision-language datasets to empower Earth Observation foundation models [33,34].

While VLMs demonstrate immense potential, their direct migration to the remote sensing domain remains highly challenging. The primary hurdle lies in the profound epistemic gap and domain shift between natural ego-centric photographs, which dominate VLM training data, and top–down overhead satellite imagery [35,36]. Extensive benchmarking has revealed that existing foundational models are often unprepared for Earth Observation data, particularly when fine-grained or pixel-level image understanding is required [37,38,39]. For example, state-of-the-art models like GPT-4V excel at high-level captioning or open-ended location understanding, but they consistently fail at tasks requiring precise counting or fine-grained spatial analysis of overhead targets [40]. When forced to analyze complex spatial arrangements, such as rotated objects, multi-level spatial contexts, or ultra-high-resolution imagery, these models frequently require additional visual prompting or extensive domain-specific datasets to mitigate their inherent spatial comprehension deficits [41,42,43].

Furthermore, current research often blindly inherits generic prompting techniques from Natural Language Processing (NLP), such as naive Chain-of-Thought (CoT) reasoning. While CoT supervision is gaining interest for tasks like visual question answering and SAR target recognition in remote sensing [44,45], and has even been introduced to multimodal small language models for small-scale remote sensing [46], applying it without domain adaptation is highly problematic. Forcing a VLM to execute step-by-step structural rationales on highly compressed, low-resolution overhead targets frequently destabilizes the classification process. This perspective disparity often leads to severe visual hallucinations, which manifest as inconsistencies between the generated text and the actual satellite image content, especially when models misinterpret macro-structures like industrial complexes or attempt to ground unobservable micro-features [47].

## 3. Methodology

**Figure 1:**
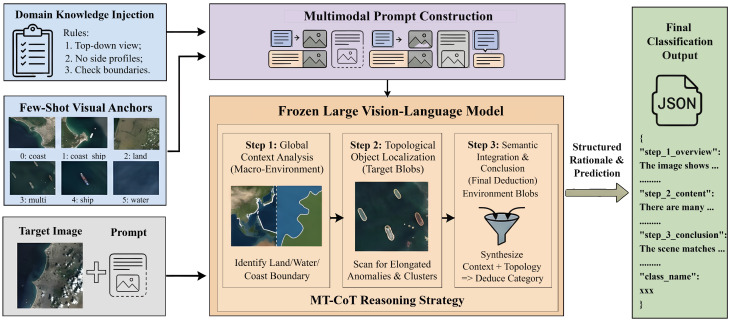
Overview of the Domain Knowledge-Enhanced In-Context Learning (DK-ICL) and Macro-Topological Chain-of-Thought (MT-CoT) Framework. The framework integrates domain knowledge rules and few-shot visual anchors into a multimodal prompt, guiding a frozen VLM through a three-step macroscopic reasoning process to achieve robust classification under extreme data scarcity.

To systematically overcome the extreme data scarcity and the inherent perspective disparity in maritime remote sensing, this paper proposes a training-free inference framework powered by VLMs, as shown in Figure 1. As illustrated in this section, we first mathematically formulate the few-shot maritime classification problem. Subsequently, we introduce the Domain Knowledge-Enhanced In-Context Learning (DK-ICL) paradigm to bridge the cross-modal epistemic gap. Finally, we detail the Macro-Topological Chain-of-Thought (MT-CoT) strategy, which orchestrates the VLM’s sequential reasoning to substantially boost target recall in complex coastal environments.

### 3.1. Problem Formulation and Task Definition

In a conventional fully-supervised maritime classification paradigm, CNNs rely heavily on a massive dataset $D_{train}={\left\{\left(x_{i},y_{i}\right)\right\}}_{i=1}^{N}$ to iteratively optimize their trainable weights θ via gradient descent. However, in time-critical MDA applications, such as disaster response or zero-day reconnaissance, acquiring large-scale annotations is fundamentally impractical, rendering *N* negligible.

To rigorously simulate this operational constraint, we formulate our task under an extreme few-shot (*K*-shot) learning setting. Let $C=\{c_{1},c_{2},...,c_{M}\}$ denote the set of macro-scene categories defined by the MASATI taxonomy (e.g., sea, land, coast, ship, multi, coast-ship). We provide the model with a minimal support set $S=\{\left(x_{k}^{\left(j\right)},y^{\left(j\right)}\right)\}$, containing exactly *K* annotated exemplars for each category *j*, where *K* is strictly limited.

We redefine the maritime scene classification task as a conditional generation problem executed with strictly frozen parameters (Δθ=0). Given a pre-trained VLM parameterized by fixed weights $\theta_{fixed}$, an unseen target optical satellite image $x_{target}$, and a meticulously engineered contextual prompt P, the model predicts the optimal scene category $\hat{y}$ by maximizing the conditional probability:

(1) $$\hat{y}=\arg\max_{y\in C}P\left(y|x_{target},S,P;\theta_{fixed}\right)$$

This training-free task definition inherently circumvents the catastrophic overfitting and computational overhead that plague traditional deep learning models when constrained to highly limited datasets. Conceptually, the mathematical viability of this formulation stems from a fundamental paradigm shift: transitioning from from-scratch feature learning to in-context adaptation. Unlike conventional supervised networks that initialize with random weights and strictly depend on massive data to carve out decision boundaries, the frozen VLM parameterized by $\theta_{fixed}$ is already equipped with a profound repository of prior knowledge acquired from billions of pre-training image–text pairs. Consequently, the minimal examples (K=4) provided in our support set S do not function as traditional training data. Instead, they act exclusively as semantic and visual triggers. Their core mechanism is to dynamically align the VLM’s vast, general-domain cognitive capabilities to the highly specific, top–down visual grammar of spaceborne marine sensors, thereby enabling robust macro-scene understanding without a single gradient update.

### 3.2. Domain Knowledge-Enhanced In-Context Learning (DK-ICL)

Off-the-shelf VLMs are intrinsically biased towards ego-centric, natural-perspective imagery acquired from extensive internet pre-training corpora. Directly querying these models with top–down spaceborne sensor imagery inevitably triggers a severe cognitive misalignment. To systematically bridge this gap without parameter fine-tuning (Δθ=0), we formalize the DK-ICL framework, which constructs a robust, multimodal contextual workspace $C_{enhance}$ prior to initiating complex reasoning.

#### 3.2.1. Formalization of the Epistemic Gap

Let $X_{nat}$ and $X_{RS}$ denote the image feature spaces of natural ego-centric photographs and overhead remote sensing imagery, respectively, while Y∈C represents the semantic category space. The VLM is predominantly optimized on the natural domain marginal distribution $P_{nat}\left(X,Y\right)$.

When inferring on spaceborne sensor imagery, the model encounters a profound epistemic gap, defined as a conditional distribution shift:

(2) $$P_{nat}\left(X|Y\right)\neq P_{RS}\left(X|Y\right)$$

Due to this severe perspective disparity, the uncalibrated posterior probability $P_{VLM}\left(Y|X_{RS}\right)$ often collapses. Therefore, our DK-ICL framework introduces a supplementary context $C_{enhance}$ to approximate the true target distribution:

(3) $$\hat{Y}=\arg\max_{Y\in C}P_{VLM}\left(Y|X_{RS},C_{enhance};\theta_{fixed}\right)$$

where the enhanced contextual workspace is formulated as the synergistic fusion of explicit domain rules and implicit visual anchors: $C_{enhance}=K_{domain}\cup S_{anchor}$.

#### 3.2.2. Heuristic Domain Knowledge Injection ($K_{domain}$)

To override the VLM’s generic semantic priors, we explicitly inject human expert interpretation heuristics into the textual prompt space. We define the domain knowledge $K_{domain}$ as a set of structured linguistic constraint functions:

(4) $$K_{domain}=\left\{f_{morph}\left(\cdot \right),f_{topo}\left(\cdot \right)\right\}$$

where:

- Morphological Constraints $f_{morph}$: The prompt structurally dictates the observation rule for vessels. (Please note: The term “morphological” here is utilized in its generic semantic sense, referring strictly to the physical shape and geometric form of the targets described as abstract concepts within the textual prompt. It does not entail the application of formal pixel-level operators from traditional Mathematical Morphology, such as erosion or dilation). Instead of searching for natural-perspective features, the model is linguistically restricted to identifying elongated topological anomalies and contrasting pixel clusters.
- Topological Relationships $f_{topo}$: The model is strictly guided to prioritize the holistic land–water boundary *B*. The spatial relationship function $f_{topo}\left(X,B\right)$ becomes the definitive criterion for distinguishing highly confusing classes, such as coast versus coast_ship.

#### 3.2.3. Few-Shot Cross-Modal Visual Anchoring ($S_{anchor}$)

Abstract textual heuristics ($K_{domain}$), while logically sound, suffer from high semantic ambiguity when processed by an uncalibrated vision encoder. To prevent semantic suspension, we redefine the *K*-shot support set S as a cross-modal visual anchoring mechanism ($S_{anchor}$).

Let the visual anchor set be defined as $S_{anchor}={\left\{\left(x_{i}^{\left(RS\right)},c_{i}\right)\right\}}_{i=1}^{K\times |C|}$, where $c_{i}$ is the class label. By mapping the structured textual rules into the visual feature space of the provided composite exemplars, the VLM establishes an instant cross-modal projection Φ:

(5) $$\Phi :K_{domain}\times S_{anchor}\to V_{aligned}$$

**Figure 2:**
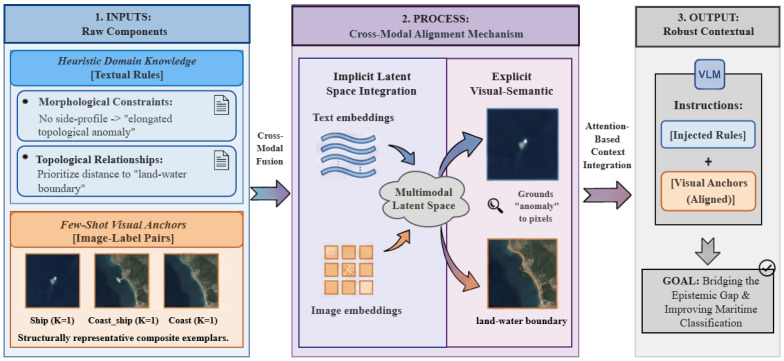
Illustration of Cross-Modal Visual Anchoring ($S_{anchor}$). The abstract textual constraint is dynamically mapped to the concrete pixel distribution of the few-shot visual anchors, effectively teaching the VLM the visual grammar of top–down overhead imagery without gradient descent.

As conceptually illustrated in Figure 2, this mechanism forces the VLM to dynamically calibrate its attention. For instance, the linguistic token “elongated anomaly” is instantly grounded to the corresponding pixel blob in the provided ship anchor. This step fundamentally neutralizes the distribution shift shown in Equation (1), teaching the VLM the visual grammar of spaceborne sensor imagery on the fly.

### 3.3. Macro-Topological Chain-of-Thought (MT-CoT)

While the DK-ICL framework establishes a robust multimodal contextual foundation ($C_{enhance}$), deploying models in complex real-world maritime scenes, especially highly confused coastal boundaries, demands a rigorous, structured reasoning pipeline. Conventional CoT prompting, typically inherited from natural language processing, encourages VLMs to search for detailed semantic features. However, applying generic CoT to highly compressed overhead imagery forces the model to guess or hallucinate unobservable micro-features (e.g., ship hull types), inevitably leading to reasoning collapse. To maximize the VLM’s inferential capacity for practical engineering deployment, we introduce the MT-CoT.

#### 3.3.1. Structured Engineering Workflow

To avoid overly complicated probabilistic reasoning paths, we redefine the CoT generation process not as free-form text generation, but as a deterministic, three-stage sequential pipeline. Unlike generic CoT where the rationale space is unbounded and prone to errors, MT-CoT explicitly decomposes the reasoning process into three practical operational states: Global Context Analysis, Topological Object Localization, and Semantic Integration. By enforcing this strict step-by-step dependency, we prevent premature semantic jumping and ensure reliable performance in real-world scenarios.

#### 3.3.2. Execution Pipeline of MT-CoT States

MT-CoT constrains the VLM to sequentially evaluate three progressive states, algorithmizing the practical cognitive process of a human remote sensing analyst.

- State 1: Global Context Analysis: Before searching for targets, the VLM first acts as a global context extractor to categorize the macroscopic environment (e.g., land, water, or coastline). This step drastically narrows the search space and generates an Environmental Manifold. In real-world deployments, this immediately prevents background industrial clutter from distracting the model.
- State 2: Topological Object Localization: Strictly conditioned on the identified environment from State 1, the VLM performs a targeted spatial scan for morphological anomalies. Instead of looking for specific “ships,” it scans for elongated blobs contrasting with the background water texture, generating a Topological Anomaly Set. This topology-first scanning mechanism is the primary catalyst for boosting target recall, preventing the model from overlooking obscure vessels.
- State 3: Semantic Integration: Finally, the deductive state acts as a logical integrator. It evaluates the spatial proximity between the localized anomalies (State 2) and the macroscopic environment (State 1) to yield the final predicted class. For instance, identifying an anomaly strictly proximate to a coastline boundary deterministically categorizes it as a coast_ship, finalizing the actionable output.

**Figure 3:**
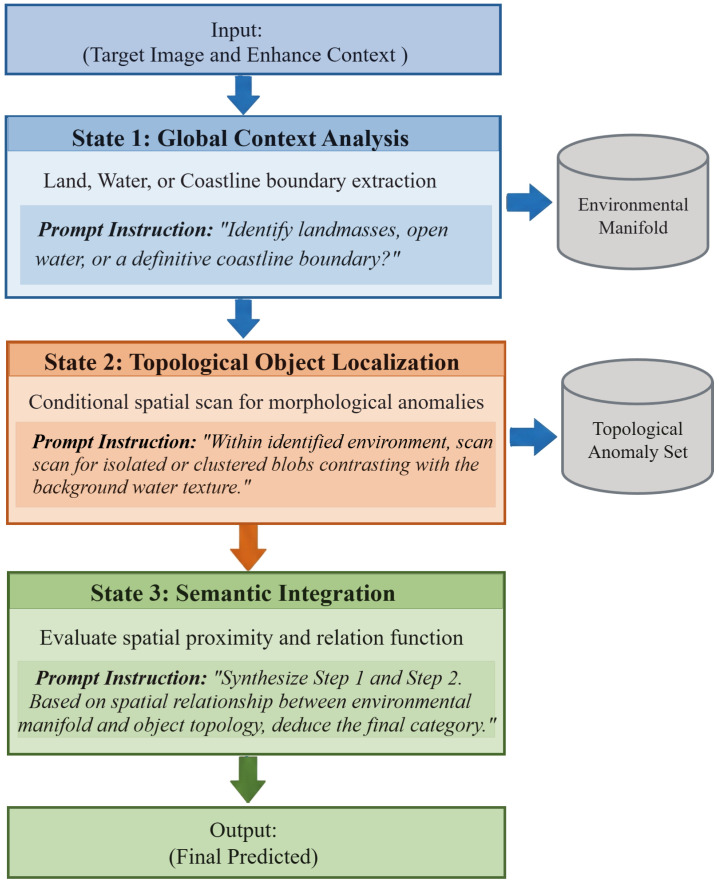
The logical execution flow of the Macro-Topological Chain-of-Thought (MT-CoT) strategy. The framework enforces a simple, strict three-step sequential pipeline: Global Context Analysis → Topological Object Localization → Semantic Integration. This structured workflow prevents resolution-induced hallucinations and ensures robust deployment in real maritime scenarios.

As visually summarized in Figure 3, the MT-CoT trajectory effectively implements these state-wise dependencies. The flowchart illustrates the structured logic: the output of the global context serves as a strict condition for anomaly localization, and the subsequent semantic step integrates both to generate the final prediction. This straightforward workflow forces the VLM to resolve spatial ambiguity sequentially, demonstrating strong implementation potential for automated maritime monitoring.

#### 3.3.3. Prompt Implementation

To operationalize this structured workflow within a frozen VLM architecture without requiring fine-tuning, we translate the three operational states into a highly explicit linguistic template. The prompt design is detailed in Table 1.

By structuring the reasoning chain around macro-topological features before attempting final classification, MT-CoT explicitly prevents the VLM from being distracted by local coastal clutter. This targeted, step-by-step constraint not only suppresses false positives along coastlines but substantially elevates the target recall rate, proving highly reliable for practical real-world applications.

## 4. Experiments and Results

To rigorously evaluate the effectiveness of the proposed DK-ICL paradigm and the MT-CoT strategy, we conducted comprehensive experiments under an extreme few-shot setting. This section details the dataset adopted, the few-shot evaluation protocol, the baseline configurations, and the quantitative metrics used to assess model performance.

### 4.1. Dataset Description

The experiments in this study are conducted on the MAritime SATellite Imagery (MASATI) dataset, a widely recognized benchmark specifically designed for maritime scene understanding and vessel detection in optical remote sensing [48]. The imagery captures top–down, overhead perspectives of complex marine and coastal environments under diverse lighting conditions, sea states, and spatial resolutions. The inherent perspective disparity and the presence of complex coastal infrastructure make it an ideal testbed for evaluating the zero-shot and few-shot reasoning capabilities of VLMs.

**Figure 4:**
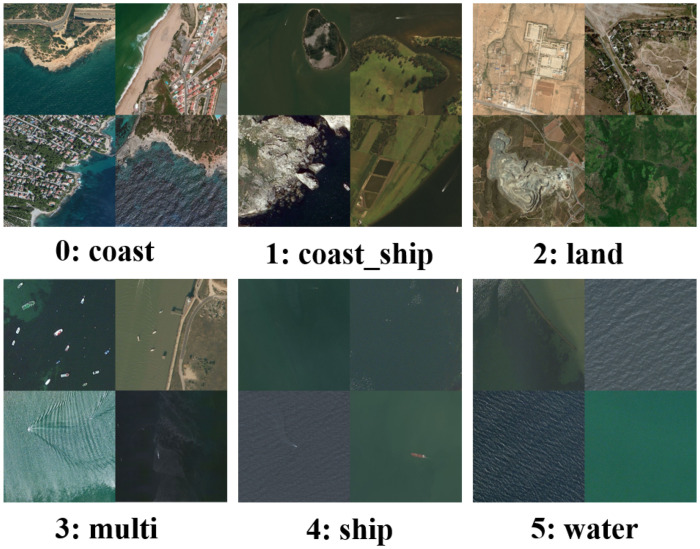
Sample composite exemplars from the restructured MASATI dataset, illustrating the six distinct macro-scene categories used for few-shot evaluation. Each category captures unique spatial topologies and environmental manifolds, presenting varying degrees of cognitive difficulty.

In our specific extreme data-scarce formulation, we systematically restructure the dataset to focus on six distinct macro-scene categories, as shown in Figure 4. These categories comprehensively cover the topological variations encountered in real-world MDA tasks. The refined taxonomy is defined as follows:

- 0: coast: Coastline or land–water boundary areas with no vessels present.
- 1: coast_ship: Coastline or land–water boundary areas containing one or more vessels (a highly challenging class prone to false positives).
- 2: land: Pure inland areas without any sea surface or water bodies.
- 3: multi: Open-water surfaces containing multiple dispersed or clustered vessels.
- 4: ship: Open-water surfaces containing a single isolated vessel.
- 5: water: Pure open-water surfaces with no vessels or landmasses present.

Extreme Few-Shot Evaluation Protocol: Unlike conventional fully-supervised deep learning approaches that consume thousands of training images to optimize model weights (θ), our framework operates in a strictly training-free regime (Δθ=0). To simulate real-world data scarcity, we adopt a *K*-shot evaluation protocol. For each experimental run, we randomly sample a microscopic support set S containing exactly *K* composite exemplars per category (K=4). These samples act exclusively as the visual anchors ($S_{anchor}$) within our multimodal prompt, rather than as gradient-descent training data. The remaining images in the dataset constitute the test set, ensuring a rigorous evaluation of the model’s pure in-context generalization and MT-CoT reasoning stability.

### 4.2. The Data Dependency Bottleneck in Traditional Architectures

To establish a rigorous baseline and empirically validate the severe data-dependency bottleneck, we systematically evaluated two representative fully-supervised architectures: ResNet-50 (a standard CNN reliant on local inductive biases) and Swin-T (a state-of-the-art Vision Transformer leveraging global self-attention). We monitored their performance trajectories as the training data scaled from an abundant regime (N=2400) down to an extreme data-scarce regime (N=24, strictly equating to K=4 shots per class).

**Figure 5:**
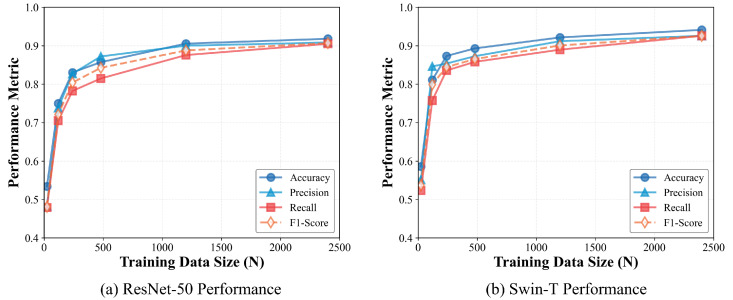
Performance degradation trajectories of traditional supervised architectures: ResNet-50 (**a**) and Swin-T (**b**). The charts illustrate the precipitous decline in all performance metrics (Accuracy, Precision, Recall, F1-Score) as the training data size (*N*) undergoes a logarithmic reduction. Notably, both models experience a severe collapse in Target Recall (red solid lines with square markers) when constrained to the extreme data-scarce regime (N=24).

The quantitative degradation of these traditional models across varying data scales is comprehensively detailed in Table 2 and visually emphasized in the dual-panel performance trajectories of Figure 5.

When provided with abundant annotated data (≥1200 samples), both architectures demonstrate robust feature extraction capabilities, achieving accuracy and F1-scores exceeding 0.90. Swin-T consistently outperforms ResNet-50 across all metrics, primarily because its hierarchical attention mechanism is better suited for capturing the global contextual manifolds of complex maritime scenes.

However, as the dataset size undergoes a logarithmic reduction, we observe a precipitous decline in model stability. The most critical observation emerges under extreme data starvation (N=24). As starkly illustrated by the sharp drop-offs in both panels of Figure 5, traditional supervised paradigms suffer severe performance degradation at this microscopic scale. Lacking sufficient statistical variance to establish generalizable decision boundaries, ResNet-50’s target recall (Figure 5, Left) plummets to a dismal 0.4789. This sub-0.50 recall indicates that the CNN’s feature space has essentially collapsed; the model resorts to near-random guessing and severely overfits to the spurious background clutter of the limited support set. Swin-T (Figure 5, Right) fares marginally better due to the stronger regularization inherent in its patch-based processing, yet it still yields an unacceptable recall of 0.5234.

These quantitative results unequivocally expose the practical limitations of conventional deep learning in time-critical scenarios. The data indicates a rigid threshold: both architectures require a minimum injection of 240 to 480 annotated samples just to recover a baseline acceptable accuracy (∼0.85). In zero-day MDA applications such as emergency disaster response or rapid target acquisition, compiling such expansive datasets is fundamentally impractical. This rigid data dependency restricts conventional models from agile deployment, clearly highlighting the engineering necessity for our training-free, VLM-driven MT-CoT paradigm.

### 4.3. Performance Evaluation Across VLM Foundation Models

Having established the severe limitations of traditional supervised paradigms under data starvation, we proceed to evaluate the efficacy of our proposed framework (DK-ICL + MT-CoT). To rigorously demonstrate that our methodology is architecturally agnostic and relies on fundamental multimodal reasoning rather than model-specific idiosyncrasies, we deploy it across a diverse spectrum of state-of-the-art Large Vision-Language Models (VLMs). These include both open-weight architectures (Qwen-3-VL, GLM-4.7) and proprietary commercial giants (Grok-4.1, GPT-4o, Gemini-3).

Crucially, all VLMs are evaluated under the exact same extreme data-scarce condition (N=24, utilized exclusively as in-context visual anchors), without undergoing any parameter fine-tuning (Δθ=0). To isolate the specific contribution of our MT-CoT framework from the general pre-trained knowledge of foundation models, we benchmark these results against traditional architectures (ResNet-50, Swin-T) as well as a pre-trained vision-language baseline (CLIP). These baselines are evaluated under both the identical scarce dataset (N=24) and a massively scaled dataset containing 50 times more annotated data (N=1200).

**Figure 6:**
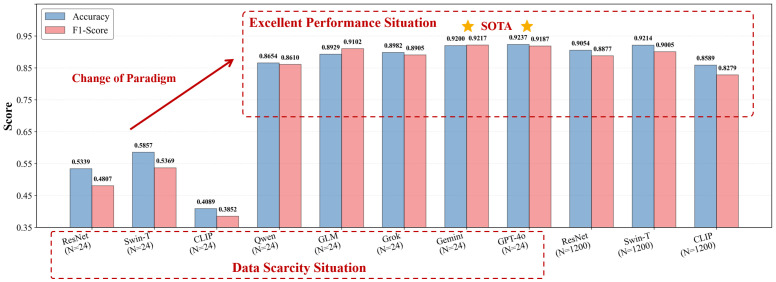
Visualizing the paradigm shift in maritime scene understanding. The bar chart compares Accuracy (blue bars) and F1-Score (red bars) across different architectures and data regimes. It highlights two critical insights: (1) Within the “Data Scarcity Situation” (N=24), the proposed VLM framework overcomes the performance collapse observed in traditional supervised models, illustrating a clear “Change of Paradigm” with up to a +38% improvement in F1-score. (2) Strikingly, top-tier VLMs utilizing only 24 samples reach the “Excellent Performance Situation,” achieving parity with, or even surpassing, data-heavy traditional models (N=1200), demonstrating that structured logical reasoning can effectively substitute massive data annotation.

The empirical results presented in Table 3 and visually emphasized in Figure 6 unequivocally validate the efficacy of our framework. A crucial observation arises when examining the CLIP baseline. Notably, simply employing a vision-language foundation model is insufficient to bridge the domain gap inherent in remote sensing. The CLIP model, despite its internet-scale multimodal pre-training, suffers a severe feature collapse under data scarcity (N=24), yielding a remarkably low F1-score of 0.3852. In stark contrast, even the relatively lightweight Qwen-3-VL, when propelled by our explicit DK-ICL and MT-CoT reasoning logic, achieves an 85.01% target recall and an F1-score of 0.8610. This definitively proves that the massive performance leap stems not merely from the VLM’s generic pre-training, but from our structured injection of domain knowledge ($K_{domain}$) that successfully translates macroscopic topological reasoning into executable logical deduction.

A fundamental transformation in maritime scene understanding emerges when comparing the data-starved VLMs (N=24) against the data-rich supervised models (N=1200). Armed with merely 4 contextual shots per class, top-tier foundation models like GPT-4o and Gemini-3 achieve F1-scores of 0.9187 and 0.9217, respectively. They not only easily eclipse the CLIP model trained on 1200 samples (F1: 0.8279), but also surpass ResNet-50 and achieve parity with the data-heavy Swin-T architecture. GPT-4o secures the highest overall accuracy (0.9237) and recall (0.9253), demonstrating a strong ability to execute the $s_{local}$ topological scanning step without missing obscure vessel targets. Meanwhile, Gemini-3 delivers the highest precision (0.9246) and F1-score, proving highly resilient against the false positives typically triggered by complex coastal backgrounds in the $s_{deduce}$ step.

Furthermore, the robust performance across the entire VLM spectrum, ranging from open-weights like GLM-4.7 to proprietary models, indicates that our DK-ICL and MT-CoT combination is universally generalizable. By offloading the burden of semantic feature learning from data-hungry weight updates to dynamic, in-context logical deduction, our framework reliably bypasses the data dependency bottleneck, enabling highly accurate deployments even in extreme zero-day scenarios.

### 4.4. Computational and Economic Cost Analysis

While Section 4.3 demonstrates the substantial performance superiority of the proposed VLM-driven MT-CoT framework, a rigorous architectural evaluation must also account for resource allocation. The paradigm shift from fully-supervised learning to training-free in-context learning introduces a fundamental trade-off between upfront sunk costs and marginal inference costs.

To systematically quantify this, we evaluate the cost profile across three critical dimensions: Data Annotation Burden (human labor), Training Compute (GPU optimization for Δθ), and Inference Overhead (per-query execution). A comprehensive structural comparison is summarized in Table 4.

Traditional supervised architectures (e.g., ResNet-50, Swin-T) rely on a highly front-loaded cost structure. Achieving an acceptable F1-score (>0.90) necessitates a massive, meticulously curated dataset (N≥1200). In the specialized domain of spaceborne sensor imagery, data annotation cannot be crowdsourced; it requires highly trained remote sensing analysts to correctly delineate complex topologies, resulting in prohibitive economic and temporal costs. Furthermore, this data must be digested through thousands of gradient descent iterations on high-performance GPU clusters, consuming substantial energy and time before the model is combat-ready. Although their eventual forward-pass inference cost is minimal, the weeks or months required for this preparation cycle strictly disqualify them from urgent response scenarios.

**Figure 7:**
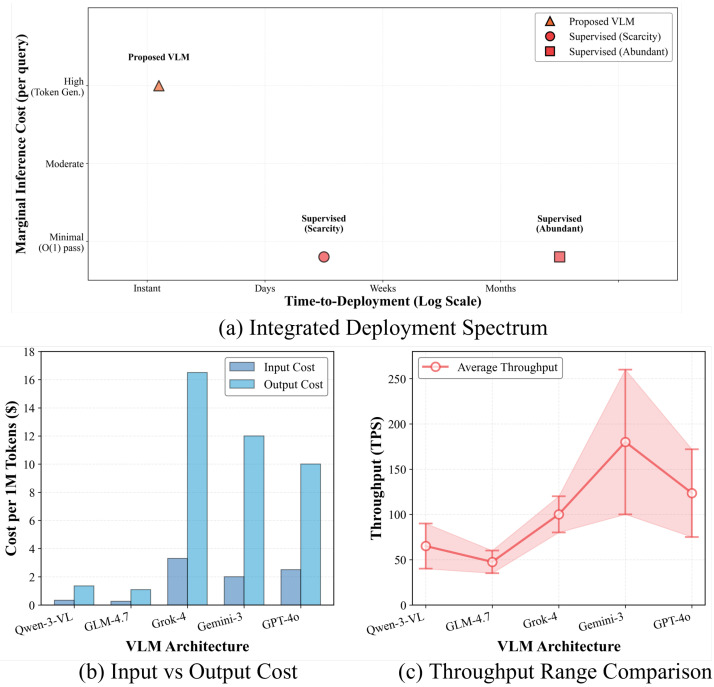
Comprehensive evaluation of deployment efficiency and economic profiling. (**a**) The Integrated Deployment Spectrum illustrates the fundamental trade-off: traditional supervised models demand significant time-to-deployment due to annotation and training, whereas the proposed VLM framework enables instantaneous deployment with zero sunk costs. (**b**) Comparison of input and output token costs per 1 million tokens across the evaluated VLMs. (**c**) Empirical throughput ranges (Tokens Per Second) demonstrating the generation speeds and operational capacities of the respective VLM architectures.

In stark contrast, as illustrated in the Integrated Deployment Spectrum (Figure 7a), our DK-ICL and MT-CoT framework radically flattens this cost curve. By operating in a strictly training-free regime (Δθ=0), the computational overhead of gradient backpropagation is entirely eliminated. The annotation burden is compressed to a negligible absolute minimum, requiring only K=4 representative exemplars to construct the visual anchor set ($S_{anchor}$).

Admittedly, the inference phase of Large Vision-Language Models (e.g., GPT-4o, Gemini-3) incurs a higher marginal computational cost per query due to the processing of dense image embeddings and the autoregressive generation of the MT-CoT reasoning tokens ($s_{global}\to s_{local}\to s_{deduce}$). However, in real-world MDA applications, the primary bottleneck is time-to-deployment, not inference compute. The ability to bypass the grueling weeks of data collection and model retraining allows our framework to be deployed instantaneously for zero-day missions. By substituting massive human annotation and GPU training hours with scalable cloud-based logical deduction, our approach presents a highly viable, agile, and economically disruptive solution for next-generation satellite intelligence.

To provide a granular understanding of the economic and temporal profiling of the specific VLMs deployed in our MT-CoT framework, Table 5 and Figure 7b,c detail the real-world API pricing and empirical performance metrics.

As delineated in Table 5 and visually compared in Figure 7b,c, the VLM ecosystem exhibits a stark bifurcation between ultimate capability and extreme cost-efficiency. Proprietary giants, specifically Gemini-3 and GPT-4o, dominate the performance frontier. Gemini-3 stands out as a computational powerhouse, boasting an unparalleled 2M context window and generating at a blistering pace of up to 260 TPS. This uniquely positions it for highly demanding future architectures, such as the simultaneous ingestion of ultra-high-resolution satellite video streams or massive spatio-temporal maritime logs. GPT-4o serves as a robust premium alternative, offering a massive 1M context with highly competitive throughput (up to 172 TPS). In contrast, while Grok-4.1 commands the highest financial premium ($16.500 per 1M output tokens), its context capacity and throughput do not establish a commensurate technological edge in this specific application domain.

On the other end of the spectrum, open-weight architectures like GLM-4.7 and Qwen-3-VL establish a highly disruptive economic paradigm. They reduce token expenditure by approximately 90% compared to their proprietary counterparts, with GLM-4.7 costing a mere $1.096 per 1M output tokens. Although their throughput ranges (35–90 TPS) are outpaced by Gemini-3, they remain more than sufficient for continuous, real-time monitoring of segmented coastal zones.

Crucially, this structural trade-off underscores the ultimate advantage of our proposed paradigm. Even when deploying the premium Gemini-3 at maximum capacity, processing thousands of satellite patches incurs merely a few dollars. When juxtaposed against the prohibitive thousands of dollars and extensive weeks required to hire human remote sensing experts for manual data curation, the token costs of our MT-CoT framework are essentially negligible. This granular profiling confirms that practitioners can flexibly interchange the VLM engine by scaling up to Gemini-3 for massive, high-throughput disaster response, or dropping down to GLM-4.7 for budget-constrained, continuous patrol, all while permanently circumventing the rigid data-dependency bottleneck of conventional architectures.

### 4.5. Ablation Study: Deconstructing the Reasoning Trajectory

To isolate and quantify the individual contributions of our proposed DK-ICL and MT-CoT modules, we conduct a rigorous ablation study. For this analytical teardown, we utilize Qwen-3-VL as the foundation model engine to demonstrate how step-wise structural prompting strictly enhances bare-metal reasoning capabilities. The evaluation is structured across three progressive configurations, all operating under the extreme data-scarce regime (K=4).

Table 6 chronicles the performance evolution from a naive zero-shot baseline to our fully integrated cognitive framework.

**Figure 8:**
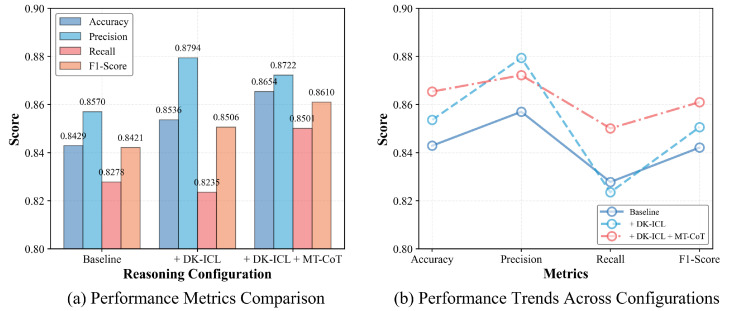
Performance evaluation of different reasoning configurations in the ablation study using Qwen-3-VL. (**a**) Absolute performance metrics comparison across the three configurations. (**b**) Performance trends illustrating the “Recall Paradox”: integrating domain anchors (+DK-ICL) increases precision but inadvertently reduces target recall due to overfitting to complex background features. The full MT-CoT framework resolves this paradox by restoring the attention mechanism, simultaneously pushing both accuracy and F1-score to their peaks.

As shown in Figure 8, the experimental outcomes reveal three critical mechanistic insights regarding VLM behavior in remote sensing:

- The Bare-Metal Baseline (Zero-Shot Capability without Examples): To transparently isolate the contribution of our 4-shot in-context examples from the VLM’s massive pre-trained knowledge, we first establish a strict zero-shot baseline (no visual examples, K=0). As shown in Table 6, the unprompted Qwen-3-VL achieves a formidable baseline accuracy of 0.8429 and an F1-score of 0.8421 solely by exploiting its billions of pre-trained image–text parameters. This zero-shot capability alone outpaces a traditional ResNet-50 trained on 240 dedicated samples (as shown previously in Table 2). However, relying purely on generic pre-trained weights leaves the model susceptible to the spatial ambiguities inherent in top–down Earth observation. The transition from this zero-shot baseline to our full DK-ICL + MT-CoT framework (which boosts the F1-score to 0.8610) empirically demonstrates the exact marginal contribution of utilizing K=4 examples as contextual adaptation triggers to overcome the epistemic gap.
- The Recall Paradox of Naive DK-ICL: By injecting the visual anchor set ($S_{anchor}$) via DK-ICL, the model acquires critical domain-specific vocabulary. Consequently, Precision surges to 0.8794, pushing overall Accuracy to 0.8536. However, as clearly depicted by the divergent trajectories in Figure 8b, we observe a counter-intuitive degradation in the Target Recall rate (dropping from 0.8278 to 0.8235). This exposes a specific vulnerability in standard few-shot prompting: without structural guidance, the VLM becomes overwhelmed by the complex background features introduced in the visual anchors. It overfits to these spurious coastal correlations, causing it to conservatively ignore elusive, partially obscured vessels (increasing False Negatives).
- MT-CoT as the Reasoning Scaffold: The integration of our MT-CoT framework effectively resolves the aforementioned bottleneck. By algorithmizing the inference into a strict macroscopic-to-topological sequence ($s_{global}\to s_{local}\to s_{deduce}$), the model is forced to explicitly localize topological anomalies before making any semantic judgments. As illustrated by the performance rebound in Figure 8b, this cognitive scaffolding elevates the overall Accuracy to 0.8654 and, crucially, restores the Recall to 0.8501 (a nearly 3% absolute gain over the DK-ICL-only setting). The resulting peak F1-Score of 0.8610 confirms that MT-CoT stabilizes the VLM’s attention mechanisms, preventing semantic hallucinations and successfully identifying complex targets that standard prompting methodologies overlook.

### 4.6. Error Analysis and Discussion: Cognitive Boundary Conditions

**Figure 9:**
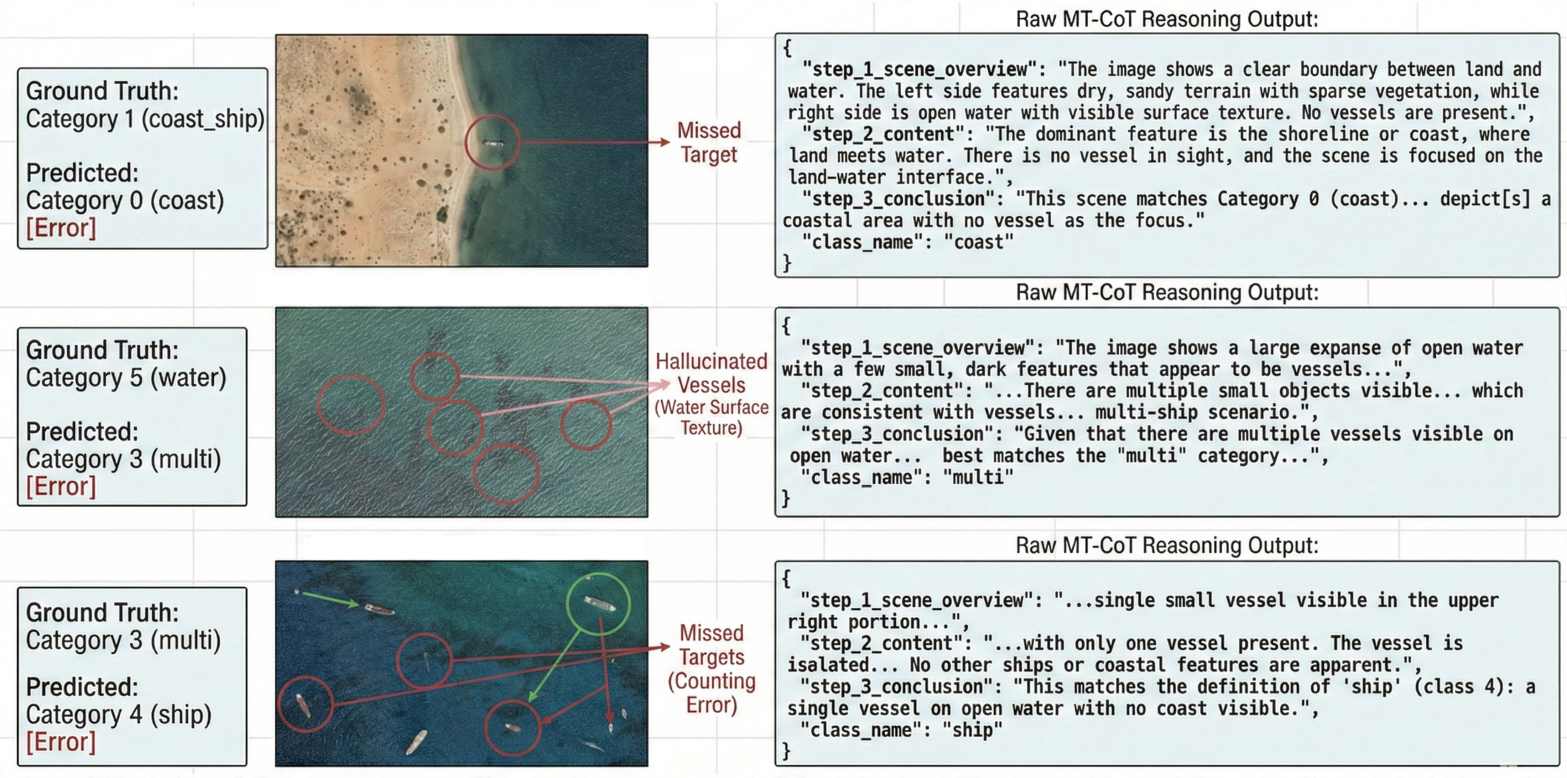
Visualizing the cognitive boundary conditions of the proposed MT-CoT framework. The diagram juxtaposes ground truth labels against VLM predictions, highlighting specific visual failures (red circles) alongside the raw autoregressive reasoning outputs. (**Top**): Resolution-to-scale omission in a complex coastal interface. (**Middle**): Semantic hallucination triggered by water surface texture. (**Bottom**): Premature search termination and counting deficit in a multi-target scenario.

While the MT-CoT framework demonstrates well in data-scarce regimes, a rigorous architectural evaluation must transparently examine its boundary conditions. To achieve this, we dissect three representative failure cases from the Qwen-3-VL inference logs. As illustrated in Figure 9, despite the structural guidance of MT-CoT, the VLM occasionally exhibits specific cognitive biases in extreme maritime scenarios.

By cross-referencing the input visual stimuli with the autoregressive textual outputs, we categorize the primary failure modes into three distinct cognitive bottlenecks:

1. Resolution-to-Scale Omission (Figure 9, Top): In this instance, a coast_ship image is misclassified as coast. The raw CoT output reveals that during $s_{global}$, the model correctly identifies the “clear boundary between land and water,” but entirely misses the extremely small vessel, concluding “No vessels are present.” This failure is driven by resolution-to-scale sensitivity. When a target occupies a minuscule pixel footprint (<1%) and is positioned adjacent to highly textured background topologies (e.g., sandy shorelines or breakwaters), the VLM’s vision encoder tends to absorb the target’s subtle feature embeddings into the dominant coastal noise, leading to a missed detection.

2. Semantic Hallucination from Surface Noise (Figure 9, Middle): Here, an empty water scene is incorrectly labeled as multi. The $s_{local}$ reasoning step explicitly hallucinates “multiple small objects visible… which are consistent with vessels.” This represents the Overfitting Trap of Topological Anomaly Scanning. Because the MT-CoT prompt aggressively forces the model to hunt for discrete topological anomalies, it can become overly sensitive to natural environmental variations. Deep wave troughs, sun glint, or cloud shadows are over-interpreted as the metallic signatures of ships. This highlights a critical trade-off: maximizing recall for elusive targets marginally increases the false positive rate in highly textured, empty oceanic zones.

3. Attentional Lock-on and Counting Deficit (Figure 9, Bottom): In this scenario, a multi vessel scene is misclassified as a single ship. The model’s reasoning exposes an attention deficit via premature search termination. The VLM successfully identifies the most salient, high-contrast target (“single small vessel visible in the upper right portion”) but subsequently terminates its spatial scanning. It completely ignores the lower-contrast, semi-obscured vessels scattered across the lower-left quadrant. This indicates that while the VLM possesses global contextual awareness, its local scanning step ($s_{local}$) lacks the exhaustive, grid-like rigor of traditional sliding-window object detectors.

These observed limitations provide a clear roadmap for future architectural refinements. To systematically address the “Resolution-to-Scale Omission,” future iterations of the MT-CoT pipeline must explicitly incorporate a Dynamic Zoom-in Mechanism. Because ultra-small targets occupying less than 1% of the pixel footprint are easily absorbed into coastal background noise, this enhancement will permit the VLM (or an external agent) to actively crop and up-sample high-entropy coastal boundaries before executing the local topological scanning step ($s_{local}$). This dynamic ‘attentional fovea’ will prevent critical target features from being diluted by macro-environments.

To mitigate semantic hallucinations, future iterations of the visual anchor set ($S_{anchor}$) must include explicit “negative anchors” (e.g., pure wave textures) to calibrate the model’s noise suppression. Finally, to resolve counting deficits, the CoT logic must be updated to enforce an explicit, quadrant-by-quadrant spatial traversal confirmation before outputting the final $s_{deduce}$ semantic judgment.

Furthermore, to systematically mitigate semantic hallucinations and resolve the “Overfitting Trap” during the anomaly scanning phase, future iterations of the framework will refine the cross-modal visual anchor set ($S_{anchor}$). As identified in our error analysis, aggressively forcing the VLM to hunt for topological anomalies can inadvertently sensitize it to natural surface variations. To counter this, we will introduce explicit “negative anchors”, specifically, visual exemplars of pure wave textures, deep wave troughs, sun glint, and cloud shadows. By directly injecting these negative visual baselines into the prompt, we can explicitly teach the VLM “what not to flag,” thereby robustly calibrating its noise suppression capabilities and significantly reducing the false-positive rate in highly textured oceanic zones.

### 4.7. Real-World Case Study: Zero-Day Deployment in Uncharted Waters

To conclusively demonstrate the practical applicability and implementation potential of our MT-CoT framework, we extend our evaluation beyond the controlled environment of the MASATI benchmark. Traditional supervised models, while performing adequately on test sets drawn from their training distribution, frequently suffer from severe generalization degradation when deployed in real-world, out-of-distribution (OOD) environments. To validate the “Zero-Day” deployment capability of our proposed framework, we conducted a real-world case study using unannotated, high-resolution optical spaceborne sensor imagery directly sourced from Google Earth.

For this operational stress test, we deliberately selected two highly complex and distinct geographical locations that were entirely unseen by the model: the Strait of Singapore (a high-density shipping lane) and the Port of Shanghai (a highly industrialized coastal zone). Crucially, the VLM was deployed instantaneously onto these raw satellite patches without a single epoch of parameter fine-tuning, relying solely on the MT-CoT reasoning pipeline.

**Figure 10:**
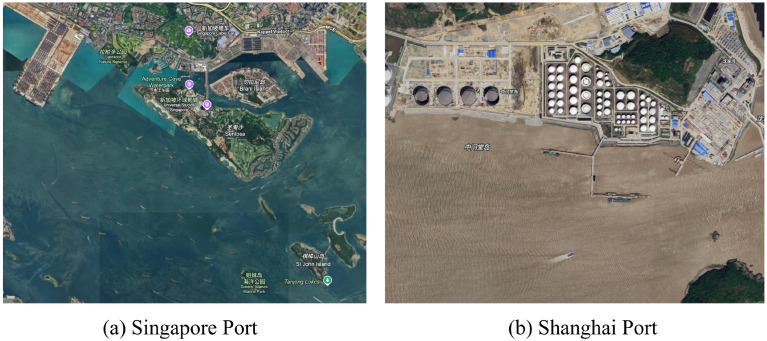
Real-world deployment case studies of the proposed MT-CoT framework using raw Google Earth imagery. (**a**): Singapore Port, demonstrating robust localization in a high-density coastal anchorage. (**b**): Shanghai Port, showcasing the model’s ability to reject severe onshore industrial clutter (e.g., storage tanks) and accurately identify moored and moving vessels in murky coastal waters. The framework successfully outputs accurate structured semantic rationales in both Zero-Day scenarios. 新加坡缆车 (Singapore Cable Car), 拉柏多公园 (Labrador Nature Reserve), 水上探险乐园 (Adventure Cove Waterpark), 新加坡环球影城 (Universal Studios Singapore), 布拉尼岛 (Brani Island), 圣淘沙 (Sentosa), 棋樟山岛 (St John Island), and 姐妹岛海洋公园 (Sisters’ Islands Marine Park).

The qualitative outcomes and the model’s internal reasoning logs are presented in Figure 10. The results strongly corroborate the robustness of the MT-CoT reasoning scaffold in practical engineering applications:

- Case A: High-Density Coastal Anchorage (Singapore Port): When scanning the Sentosa island region, the model faced an extreme density of targets. Following the MT-CoT pipeline, the VLM first accurately established the global context (Step 1), identifying “distinct islands and port infrastructure” interfacing with “open water.” Strictly within this water manifold, the targeted spatial scanner (Step 2) successfully identified a “high density of isolated and clustered elongated blobs” despite the dark water texture. Finally, the semantic integration (Step 3) logically deduced that this spatial relationship indicates a “high-traffic coastal anchorage,” correctly classifying the scene as multi. This demonstrates the framework’s scalability in handling massive target volumes without cognitive collapse.
- Case B: Complex Industrial Coastline (Shanghai Port): The Shanghai port scenario presents a severe challenge due to murky water textures and dense onshore industrial infrastructure (e.g., massive cylindrical storage tanks) that frequently trigger false positives in generic CNNs. The MT-CoT framework exhibited exceptional operational precision here. By anchoring the reasoning to the “definitive coastline” (Step 1), the model completely ignored the onshore oil tanks. Instead, it localized specific topological anomalies (Step 2) “positioned alongside pier structures” and a “moving object… displaying a visible wake” in the murky water. The deduction phase (Step 3) synthesized this coastal industrial context to definitively confirm the presence of multiple maritime vessels (multi).

These real-world deployment tests confirm that substituting generic text generation with a structured, macro-topological reasoning pipeline successfully prevents resolution-induced hallucinations. By grounding inferences strictly in topological facts and environmental boundaries, the MT-CoT framework provides a highly reliable, zero-gradient solution for automated maritime monitoring in uncharted environments.

## 5. Discussion

### 5.1. From Weight Optimization to Contextual Engineering

For the past decade, the dominant paradigm in Earth Observation has been fully-supervised gradient descent. Researchers have treated neural networks as ’blank slates,’ requiring massive, exhaustively annotated datasets to painstakingly carve domain-specific decision boundaries into the model weights (Δθ). Our DK-ICL and MT-CoT framework explicitly challenges this necessity. By demonstrating that a frozen Vision-Language Model (VLM) can achieve parity with, or even surpass, data-heavy traditional architectures using merely K=4 shots per class, we validate a transition from weight optimization to contextual engineering. The VLM already possesses the requisite cross-modal cognitive capacity; the challenge is no longer about teaching the model ’what’ a ship is, but rather guiding its vast prior knowledge to adapt to the top–down perspective of optical satellite sensor data through structured logical scaffolding.

While the broader machine learning community has extensively explored the integration of foundation models to compensate for limited training data, their direct application to Earth Observation is frequently hindered by a profound epistemic gap. Therefore, the contribution of our DK-ICL and MT-CoT framework is not a fundamental architectural invention within the VLM itself, but rather a targeted contextual engineering paradigm. It serves as the crucial translational bridge, demonstrating how generic cross-modal cognitive capacities can be successfully operationalized for highly specialized, top–down maritime scene understanding.

### 5.2. Economic Viability and ’Zero-Day’ MDA Deployment

In real-world MDA applications, such as disaster response, illegal fishing monitoring, or military reconnaissance, time is the most critical asset. Traditional deep learning pipelines are inherently delayed by the weeks or months required for data curation, expert annotation, and GPU cluster training. As quantified in our cost analysis, the VLM-driven approach entirely bypasses these prohibitive sunk costs. While API token generation incurs a marginal inference fee, it offers unparalleled agility. The ability to deploy a highly accurate, robust monitoring system instantaneously presents a highly disruptive economic and operational advantage over conventional methodologies.

### 5.3. Integration with Multi-Sensor Maritime Networks

While this study primarily focuses on optical sensor data, the proposed MT-CoT framework demonstrates significant potential for broader sensing technology applications. In modern marine vehicle systems and coastal monitoring networks, decision-making relies on heterogeneous sensor fusion. Future iterations of our VLM-driven paradigm can act as a central cognitive processing hub, actively fusing visual inputs from optical/infrared camera sensors with numerical data from Automatic Identification System (AIS) sensors and shipborne navigation sensors. By translating raw, multi-modal sensor streams into structured semantic deductions without the need for extensive sensor-specific retraining, this paradigm substantially reduces equipment operation and maintenance costs, paving the way for next-generation intelligent sensing arrays in maritime environments.

### 5.4. Towards Agentic Remote Sensing and Robust Refinement

While the MT-CoT framework significantly mitigates the VLM’s inherent resolution sensitivity and semantic hallucinations, it also paves the way for fully autonomous “Agentic” remote sensing workflows. Future research should focus on embedding these VLMs within tool-using agent frameworks to systematically address current cognitive boundary conditions. To resolve the “Resolution-to-Scale Omission,” we propose the integration of a Dynamic Zoom-in Mechanism. This would permit the VLM to actively crop and up-sample high-entropy coastal regions before executing local topological scans ($s_{local}$), effectively functioning as a dynamic “attentional fovea” that prevents small vessels from being absorbed into background noise.

Furthermore, to mitigate the “Overfitting Trap” and eliminate semantic hallucinations triggered by water surface texture, future iterations will refine the visual anchor set ($S_{anchor}$) by introducing explicit “negative anchors.” By directly injecting visual exemplars of pure wave troughs, sun glint, and cloud shadows into the multimodal prompt, we can explicitly teach the VLM “what not to flag.” This calibrated noise suppression will significantly reduce false positives in highly textured oceanic zones. By translating raw, multi-modal sensor streams into structured semantic deductions and autonomously triggering these refinement tools, this paradigm transforms static image classifiers into interactive maritime intelligence analysts.

### 5.5. Limitations

While the DK-ICL and MT-CoT framework demonstrates overwhelming potential for data-scarce maritime scene understanding, a rigorous scientific evaluation necessitates acknowledging several inherent limitations within the current paradigm:

- Pre-training Bias and Reproducibility: The framework relies heavily on off-the-shelf VLMs that are predominantly pre-trained on natural, ego-centric internet data. This profound reliance introduces a potential risk of uncontrolled cognitive biases when models attempt to generalize to out-of-distribution spaceborne sensor data. Furthermore, while open-weight models (e.g., Qwen-3-VL) show strong promise, the absolute peak “zero-day” performance currently relies on proprietary models (e.g., GPT-4o, Gemini-3). The opaque nature of their architectures and inaccessible training corpora inherently limits strict scientific reproducibility.
- Prompt Engineering and Domain Transferability: The MT-CoT strategy demands non-trivial, highly specialized prompt engineering. The structural text and macro-topological constraints are meticulously tailored to the maritime domain. Consequently, this specific pipeline may not seamlessly transfer to other remote sensing domains (e.g., urban planning or agricultural monitoring) without substantial, domain-specific adaptation and expert heuristics.
- Anchor Sensitivity: The pipeline exhibits notable sensitivity to the selection of the few-shot visual anchors ($S_{anchor}$). Currently, the framework relies on heuristically selected, representative exemplars. The absence of a systematic, automated selection procedure implies that suboptimal anchor choices could destabilize the cross-modal alignment and degrade inference reliability.
- Resolution Boundaries: As extensively diagnosed in our error analysis (Section 4.6), the framework possesses an intrinsic cognitive difficulty in managing ultra-small targets (e.g., those occupying less than 1% of the pixel footprint). Without external dynamic zoom-in mechanisms, these sub-threshold targets are easily absorbed into complex coastal background noise.
- Inference Cost in Continuous Monitoring: Finally, although our training-free paradigm successfully circumvents the massive upfront sunk costs of data annotation and GPU training, the token-based execution structure presents a different economic bottleneck. The marginal computational inference costs (API token fees and latency) can accumulate significantly, making it potentially cost-prohibitive for ultra-high-frequency, continuous global monitoring scenarios compared to the lightweight forward passes of traditional CNNs.

### 5.6. Deployment Architecture and Operational Requirements

To transition the proposed DK-ICL and MT-CoT framework from a theoretical proof-of-concept into “next-generation intelligent maritime sensing networks,” it is imperative to define the practical deployment architecture. Given the extreme operating conditions of maritime environments, we propose a Hybrid Edge-Cloud Deployment Paradigm, which bifurcates based on model accessibility and mission criticality:

Cloud-Centric Deployment (Ground Station APIs): For proprietary foundation models offering peak “zero-day” performance (e.g., GPT-4o, Gemini-3), execution cannot occur locally on maritime platforms. Instead, the architecture dictates that spaceborne or shipborne optical sensors downlink compressed image patches to terrestrial ground stations via maritime satellite communications (SATCOM, e.g., VSAT or Starlink). However, this introduces severe connectivity constraints. Maritime bandwidth is often intermittent and limited, introducing unavoidable latency during the uplink/downlink cycle. More critically, transmitting raw sensor data to commercial cloud APIs presents significant security and data privacy risks, rendering this cloud-dependent approach unsuitable for militarily sensitive or mission-critical reconnaissance operations.

Edge-Centric Deployment (Shipboard Processing): To circumvent network dependency and guarantee data sovereignty, the framework strongly advocates for deploying open-weight VLMs (e.g., Qwen-3-VL, GLM-4.7) directly on localized edge devices, such as shipboard processing servers or autonomous surface vehicle (ASV) compute payloads.

Hardware Prerequisites and Optimization: Deploying a standard 7B to 8B parameter VLM in half-precision (FP16) typically requires 16 to 24 GB of Video RAM (VRAM) and substantial computing power (TFLOPS). To achieve viability on maritime embedded systems, models must be subjected to structural quantization (e.g., INT8 or INT4), which drastically reduces the memory footprint to under 8 GB. Modern edge AI platforms, such as the NVIDIA Jetson AGX Orin, possess sufficient unified memory and low power consumption profiles (ranging from 15 W to 60 W) to host these quantized VLMs. Under such configurations, empirical throughput can be stabilized to process local image patches in near-real-time, providing fully “air-gapped,” highly secure, and zero-latency maritime scene understanding without any reliance on external internet connectivity.

## 6. Conclusions

This paper addresses the critical bottleneck of extreme data scarcity in MDA by proposing a novel, training-free inference paradigm. As traditional supervised architectures suffer catastrophic feature collapse under severely limited data constraints, our research demonstrates that VLMs can successfully bypass the prohibitive costs of gradient-based weight optimization. By introducing the DK-ICL framework coupled with a MT-CoT strategy, we systematically bridged the epistemic gap between ego-centric natural images and top–down spaceborne sensor imagery.

Extensive empirical evaluations confirm the superiority of this approach. Using merely 4 unoptimized visual exemplars per category, the MT-CoT augmented VLMs outperformed traditional models trained under identical scarcity by over 38% in F1-score, achieving performance parity with data-heavy networks trained on 50 times the data volume. Ultimately, by substituting massive manual annotation and GPU training with scalable, cloud-based logical deduction, our framework establishes a highly viable foundation for instantaneous “Zero-Day” deployment in next-generation intelligent maritime sensing networks. The seamless integration of optical sensor arrays and VLM-driven cognitive analytics will profoundly empower the shipping industry. It transforms traditional, passive sensor data collection into an agile, autonomous, and highly resilient maritime monitoring system.

Moving forward, future research will focus on addressing the cognitive boundary conditions identified in our empirical analysis. We plan to integrate a dynamic zoom-in mechanism to mitigate resolution-to-scale omissions for ultra-small targets, and explore multi-sensor fusion strategies (e.g., combining optical sensor data with AIS and shipborne radar sensors) to further suppress semantic hallucinations in highly textured waters.

## Figures and Tables

**Table 1:** The Structural Prompt Template of MT-CoT.

System Role: You are an expert maritime intelligence analyst specializing in optical spaceborne sensor imagery.
Domain Knowledge ($K_{domain}$): Note that satellite images are top–down overhead views. Ships lack side profiles and appear as elongated topological anomalies on water surfaces. Pay strict attention to the holistic land–water boundaries.
Visual Anchors ($S_{anchor}$): [Provided *K*-shot composite exemplars mapping to the MASATI categories]
MT-CoT Reasoning Instructions:
Execute the following reasoning steps strictly based on macroscopic topologies:
Step 1 (Global Context Analysis): Does the image contain landmasses, open water, or a definitive coastline boundary? (This narrows the environmental candidate space).
Step 2 (Topological Object Localization): Within the identified environment, scan for topological anomalies. Are there isolated or clustered elongated blobs contrasting with the background water texture? (This exhaustive scan boosts target recall).
Step 3 (Semantic Integration): Synthesize Step 1 and Step 2. Based on the spatial relationship between the macroscopic environment and the object topology, deduce the final category.
Output Format: Output a structured JSON containing: {“Step_1”: “…”, “Step_2”: “…”, “Step_3”: “…”, “class_id”: <int>}

**Table 2:** Performance Degradation of Traditional Supervised Models across Varying Training Data Sizes.

Size (*N*)	ResNet-50				Swin-T			
	**Acc**	**Precision**	**Recall**	**F1**	**Acc**	**Precision**	**Recall**	**F1**
24	0.5339	0.4826	0.4789	0.4807	0.5857	0.5512	0.5234	0.5369
120	0.7500	0.7382	0.7052	0.7213	0.8107	0.8460	0.7575	0.7993
240	0.8304	0.8271	0.7822	0.8040	0.8732	0.8535	0.8354	0.8443
480	0.8571	0.8720	0.8147	0.8424	0.8929	0.8726	0.8579	0.8652
1200	0.9054	0.9000	0.8756	0.8877	0.9214	0.9116	0.8896	0.9005
2400	0.9179	0.9083	0.9048	0.9066	0.9411	0.9257	0.9256	0.9256

**Table 3:** Comprehensive Quantitative Comparison. The proposed VLM-driven framework (using only 24 un-optimized support samples) is benchmarked against traditional supervised models and a CLIP vision-language baseline trained under both identical scarcity (N=24) and abundant data regimes (N=1200). Bold values indicate the best performance.

Model Architecture	Data (*N*)	Acc	Precision	Recall	F1
ResNet-50	24	0.5339	0.4826	0.4789	0.4807
	1200	0.9054	0.9000	0.8756	0.8877
Swin-T	24	0.5857	0.5512	0.5234	0.5369
	1200	0.9214	0.9116	0.8896	0.9005
CLIP	24	0.4089	0.4333	0.3468	0.3852
	1200	0.8589	0.8838	0.7787	0.8279
Qwen-3-VL	**24**	0.8654	0.8722	0.8501	0.8610
GLM-4.7	**24**	0.8929	0.9295	0.8918	0.9102
Grok-4.1	**24**	0.8982	0.9068	0.8747	0.8905
Gemini-3	**24**	0.9200	**0.9246**	0.9189	**0.9217**
GPT-4o	**24**	**0.9237**	0.9123	**0.9253**	0.9187

**Table 4:** Comparative Analysis of Computational and Economic Costs. The proposed VLM framework completely eliminates the massive sunk costs of data annotation and model training, trading them for a higher, yet highly scalable, token-based inference cost, thereby enabling zero-day deployment.

Cost Dimension	Supervised (Abundant)	Supervised (Scarcity)	Proposed VLM
Annotation Burden	Massive (N≥1200)	Low (N=24)	Negligible (K=4)
Training Compute (Δθ)	High (GPU Days)	Moderate (GPU Hours)	Zero (Δθ=0)
Inference Cost	Minimal (O(1) pass)	Minimal (O(1) pass)	High (Token Gen.)
Time-to-Deployment	Weeks/Months	Days (Fails in Accuracy)	Instantaneous (Zero-Day)

**Table 5:** Empirical Inference Cost, Context Capacity, and Throughput Profiling across Evaluated VLMs. Economic costs are standardized per 1 Million (1M) tokens based on prevailing API structures. Throughput (Tokens Per Second, TPS) ranges represent empirical generation speeds, serving as a critical metric for large-scale maritime deployments.

VLM Architecture	Context Window	Input Cost (1 M)	Output Cost (1 M)	Throughput (TPS)
Qwen-3-VL	256 K	$0.342	$1.369	40–90
GLM-4.7	200 K	$0.274	$1.096	35–60
Grok-4.1	256 K	$3.300	$16.500	80–120
Gemini-3	2 M	$2.000	$12.000	100–260
GPT-4o	1 M	$2.500	$10.000	75–172

**Table 6:** Ablation Study on the Qwen-3-VL Architecture. The table tracks the quantitative impact of progressively injecting DK-ICL and MT-CoT. Bold values indicate the optimal performance across the configurations.

Reasoning Configuration	Accuracy	Precision	Recall	F1-Score
Baseline (Direct Zero-Shot)	0.8429	0.8570	0.8278	0.8421
+ DK-ICL (Domain Knowledge Anchors)	0.8536	**0.8794**	0.8235	0.8506
+ DK-ICL + MT-CoT (Full Framework)	**0.8654**	0.8722	**0.8501**	**0.8610**

## Data Availability

The data presented in this study are openly available in https://www.iuii.ua.es/datasets/masati/, accessed on 16 April 2026.
